# Nonstoichiometry Defects in Double Oxides of the A_2_BO_4_-Type

**DOI:** 10.3390/ma15217642

**Published:** 2022-10-31

**Authors:** Aleksandr S. Gorkusha, Sergey V. Tsybulya, Svetlana V. Cherepanova, Evgeny Y. Gerasimov, Svetlana N. Pavlova

**Affiliations:** 1 Boreskov Institute of Catalysis SB RAS, 630090 Novosibirsk, Russia; 2 Department of Physics, Novosibirsk State University, 630090 Novosibirsk, Russia

**Keywords:** Ruddlesden–Popper phases, X-ray powder diffraction, HRTEM, defects, 1D simulation, nonstoichiometry, oxidative coupling of methane

## Abstract

Double oxides with the structure of the Ruddlesden–Popper (R-P) layered perovskite A_n+1_B_n_O_3n+1_ attract attention as materials for various electrochemical devices, selective oxygen-permeable ceramic membranes, and catalytic oxidative reactions. In particular, Sr_2_TiO_4_ layered perovskite is considered a promising catalyst in the oxidative coupling of methane. Our high-resolution transmission electron microscopy (HRTEM) studies of Sr_2_TiO_4_ samples synthesized using various methods have shown that their structure often contains planar defects disturbing the periodicity of layer alternation. This is due to the crystal-chemical features of the R-P layered perovskite-like oxides whose structure is formed by n consecutive layers of perovskite (ABO_3_)_n_ in alternating with layers of rock-salt type (AO) in various ways along the **c** crystallographic direction. Planar defects can arise due to a periodicity violation of the layers alternation that also leads to a violation of the synthesized phase stoichiometry. In the present work, a crystallochemical analysis of the possible structure of planar defects is carried out, structures containing defects are modeled, and the effect of such defects on the X-ray diffraction patterns of oxides of the A_2_BO_4_ type using Sr_2_TiO_4_ is established as an example. For the calculations, we used the method of constructing probabilistic models of one-dimensionally disordered structures. For the first time, the features of diffraction were established, and an approach was demonstrated for determining the concentration of layer alternation defects applicable to layered perovskite-like oxides of the A_2_BO_4_ type of any chemical composition. A relation has been established between the concentration of planar defects and the real chemical composition (nonstoichiometry) of the Sr_2_TiO_4_ phase. The presence of defects leads to the Ti enrichment of particle volume and, consequently, to the enrichment of the surface with Sr. The latter, in turn, according to the data of a number of authors, can serve as an explanation for the catalytic activity of Sr_2_TiO_4_ in the oxidative coupling of methane.

## 1. Introduction

Complex oxides of the general formula A_n+1_B_n_O_3n+1_ with the layered Ruddlesden–Popper (R-P) structure attract considerable attention of researchers as promising materials for electrochemical and magnetic devices, selective oxygen-conducting membranes and also as catalysts for various processes [1,2,3,4]. In particular, oxides of the R-P series (with the compositions A = Ca, Ba, Sr, B = Ti, Sn) are considered promising catalysts for the oxidative coupling of methane (OCM) [5,6,7,8,9,10], a one-stage method for producing ethane and ethylene (C2), for which an active search for effective catalysts is currently underway [11,12,13]. It is believed that the features of the R-P structure, which consists of alternating blocks with structures of the ABO_3_ perovskite-type and the AO rock salt type [14], determine the high activity and selectivity of catalysts in OCM [9,10]. Earlier in [15], we showed that in single-phase or containing a minimum amount of impurity phases, Sr_2_TiO_4_ samples could be obtained using a synthesis procedure that includes the stages of mechanochemical activation of precursors and high-temperature calcination.

However, the structural features of layered perovskites allow the presence of various types of defects, and the real structure of the resulting phases can be the cause of their specific physical and/or chemical (including catalytic) properties.

Nonstoichiometry defects in the structure of R-P oxides have been previously the subject of a large number of experimental and theoretical studies [16,17,18,19,20,21].

It has been repeatedly shown by HRTEM that extended (planar) defects can occur in these structures, violating the periodicity in the alternation of layers [19,20].

In particular, our preliminary studies [15,22] also showed that the structure of some Sr_2_TiO_4_ samples obtained using the mechanochemical activation of precursors contains planar defects, leading to nonstoichiometry of the formed phases both in terms of the ratio of cations and oxygen content. In principle, quantitative estimates of the content of planar defects can be obtained from X-ray powder diffraction data if a relationship is established between the presence of planar defects of one type or another and changes in the diffraction patterns [23]. At present, there is no such analysis for structures of the A_2_BO_4_ type.

In this paper, we consider the influence of planar defects associated with the violation of the order in the alternation of layers in structures of the A_2_BO_4_ type (using Sr_2_TiO_4_ as an example) on their diffraction patterns and propose a method for estimating the concentration of defects from X-ray powder diffraction data.

## 2. Materials and Methods

### 2.1. Sample Synthesis

The samples under study were selected from a series of samples synthesized in [15].

As the starting materials, SrCO_3_, TiO_2_ (rutile), and TiO(OH)_2_ were used to prepare the Sr_2_TiO_4_ samples. To provide a target stoichiometry of samples, corresponding amounts of starting compounds were taken on the basis of their thermal analysis. The stoichiometric mixtures of the starting chemicals were mixed and then activated in two modes.

In the first mode (sample No. 1), an APF-5 low-energy mill was used. The starting compounds are SrCO_3_ and TiO(OH)_2_. Activation was carried out with zirconium balls 5 mm in diameter in water-cooled iron drums with a volume of 25 mL. The ratio of the mass of the balls to the mass of the mixture was 10, the drum rotation speed was 800 rpm, and activation was carried out within 10 min.

In the second mode (sample No. 2) of activation, a high-voltage planetary mill AGO-2 was used. The starting compounds are SrCO_3_ and TiO_2_. Activation was carried out with zirconium balls 5 mm in diameter in water-cooled iron drums with a volume of 150 mL. The ratio of the mass of the balls to the mass of the mixture was 20, the drum rotation speed was 1200 rpm, and activation was carried out for 20 min.

Before each synthesis, a preliminary treatment of the drums and balls with the corresponding mixture was performed to cover the surface of the drums and balls with a layer of the initial mixture. This minimizes the contamination of the samples with Fe and Zr due to their rubbing during mechanochemical activation. 

Activated mixtures were pressed in tablets and annealed at 1100 °C for 4 h. 

The choice of samples with different backgrounds was conscious. On the one hand, the chosen samples were almost single-phase; on the other hand, they had differences in the real structure, as shown below.

### 2.2. Sample Characterization

#### 2.2.1. XRD

Diffraction experiments were performed using synchrotron radiation (λ = 1.5369 Å) in the Bragg–Brentano geometry at the Siberian Center for Synchrotron and Terahertz Radiation [24]. The refinement of the averaged crystal structure, including the lattice parameters and diffraction patterns, was analyzed using the Rietveld method using the GSAS-II program [25].

Simulation of diffraction patterns in the presence of layer alternation defects was performed according to the method described in detail in the monograph [26]. The diffraction patterns were calculated based on a statistical model of a one-dimensional (1D) disordered crystal. The calculations use the fact that the scattering region from a two-dimensional (2D) periodic layer is localized along the rods passing through the nodes of the two-dimensional periodic reciprocal lattice, which are determined by integers h and k. The same localization of the scattering region is also preserved for a one-dimensional disordered crystal. The model of a 1D-disordered crystal is presented as a statistical sequence of two-dimensional periodic layers of various types. As a rule for generating a statistical sequence of layers, a Markov chain of the nth order is used. The zero-order S = 0 means a completely random sequence of layers. A Markov chain with an order greater than zero is used to set the short-range order in the alternation of layers or in the methods of their superposition methods. In our calculations, we used the first-order Markov chain (S = 1), which makes it possible to create both strictly ordered structures and structures with layer alternation defects.

#### 2.2.2. HRTEM

The structure and microstructure of the samples were studied by HRTEM using a ThemisZ electron microscope (Thermo Fisher Scientific, Waltham, MA, USA) with an accelerating voltage of 200 kV and a limiting resolution of 0.07 nm. Images were recorded using a Ceta 16 CCD array (Thermo Fisher Scientific, USA). High-angle annular dark-field imaging (HAADF STEM image) was performed using a standard ThemisZ detector. The instrument is equipped with a SuperX (Thermo Fisher Scientific, USA) energy-dispersive characteristic X-ray spectrometer (EDX) with a semiconductor Si detector with an energy resolution of 128 eV.

For electron microscope studies, sample particles were deposited on perforated carbon substrates fixed on copper grids using a UZD-1UCH2 ultrasonic disperser, which made it possible to achieve a uniform distribution of particles over the substrate surface. The sample is placed in an alcohol drop which is deposited on an ultrasonic disperser. After an increase in the ultrasonic frequency, the mixture of alcohol and sample in the form of vapor falls onto a standard copper grid.

Fast Fourier Transform Images (FFT—images) were made in Digital Micrograph software (Gatan, Pleasanton, CA, USA) from a selected area in the HRTEM images.

## 3. Results and Discussion

### 3.1. Interpretation of Experimental Data

#### 3.1.1. X-ray Diffraction Analysis

**Figure 1:**
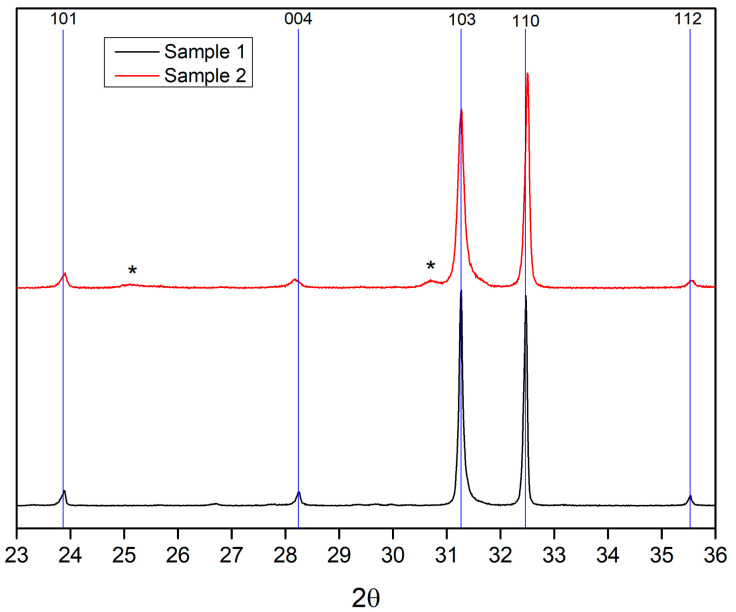
Comparison of the X-ray diffraction patterns of samples 1 and 2. The lines show the positions of the Sr_2_TiO_4_ reflections for sample 1. Asterisks indicate the weak peaks of impurity phases.

**Figure 2:**
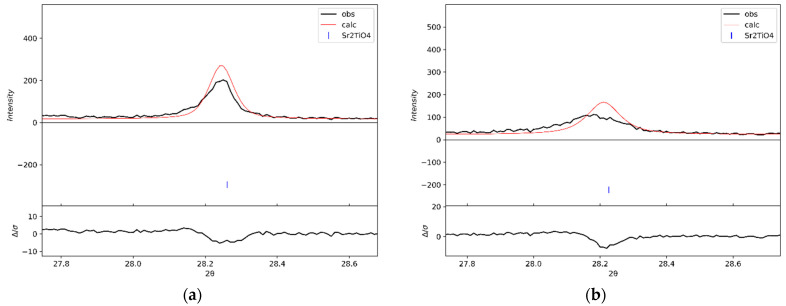
Positions of the experimental and calculated 004 XRD peaks based on the results of the refinement of the sample structures by the Rietveld method. (**a**) Sample 1; (**b**) Sample 2.

According to the preliminary X-ray phase analysis (Figure 1), samples 1 and 2 were practically single-phase layered Sr_2_TiO_4_ perovskites. Small peaks of impurity phases are marked with asterisks: a peak at about 25.1° corresponds to an interplanar distance of 3.53 Å and is probably associated with the strontium carbonate phase [PDF 05-0418]; a peak with position 30.8° was not identified, unfortunately. However, a more detailed comparison of the X-ray diffraction patterns (Figure 1) showed that they are not identical—the positions of some reflections of the two samples differ (see the bar diagram in Figure 1). In addition, the peaks in the X-ray diffraction pattern of sample 2 are noticeably broader. The ratios of the peak heights for the samples also differ somewhat, which is especially noticeable for the pair of the strongest reflections, 103 and 110. The crystal structure models were refined by the Rietveld method (Figure S1a,b, Table S1). But for sample 2, some experimental peaks are shifted relative to the positions calculated from the refined average values of the lattice parameters. This effect is especially noticeable for peak 004 (Figure 2). The observed differences in the diffraction patterns of the two samples can be associated with the features of the real structure of the Sr_2_TiO_4_ synthesized. Therefore, we carried out studies using HRTEM.

#### 3.1.2. Electron Microscopy

**Figure 3:**
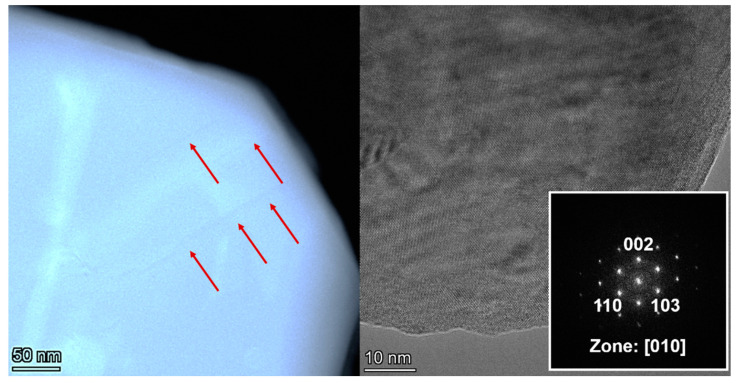
HAADF-STEM and HRTEM images of sample 1. Red arrows show planar defects in [001] direction, white square inset shows the corresponding FFT image.

**Figure 4:**
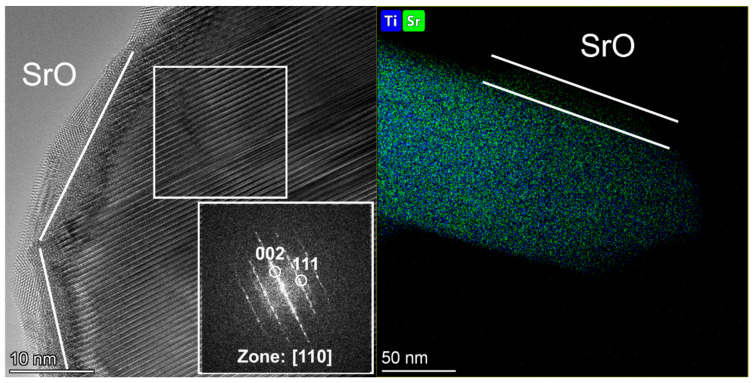
HRTEM image and EDX mapping of sample 2. The white square inset shows the corresponding FFT image with planar defects in [001] directions.

HRTEM data have shown that both samples exhibit characteristic planar defects disrupting the structure periodicity and layers alternation in the [001] direction (Figure 3 and Figure 4), but they are more numerous in sample 2. Similar results for systems with a perovskite structure were observed in [27,28].

The HRTEM data also showed that the surface of the particles is covered with an oxide shell different from the main phase. Measurements of the interplanar distances of the coating showed that the oxide shell corresponds to SrO. It can be formed as a result of the incomplete decomposition of hydrocarbonate phases or SrCO_3_ (traces of which are detected by XRD, Figure 1) due to the action of an electron beam on the sample under study in the microscope chamber. A detailed study of the reasons for the formation of such heterogeneous systems is presented in [29].

Partial segregation of divalent cations (Sr, Ca, etc.) on the surface due to the mechanisms proposed in [29] causes the formation of vacancy structures in the perovskite matrix that could lead to the formation of planar defects, both in the ABO_3_ [30] and in the R-P structures [20]. This effect can arise due to the structural features of perovskite-like oxides as a result of the synthesis of complex oxides and their subsequent processing.

What can be the reason for the existence of such defects in Sr_2_TiO_4_? Planar defects associated with a violation of the order of alternation of layers can arise due to the peculiarities of the Ruddlesden–Popper structural series.

#### 3.1.3. Structural Features of the Ruddlesden–Popper Series

**Figure 5:**
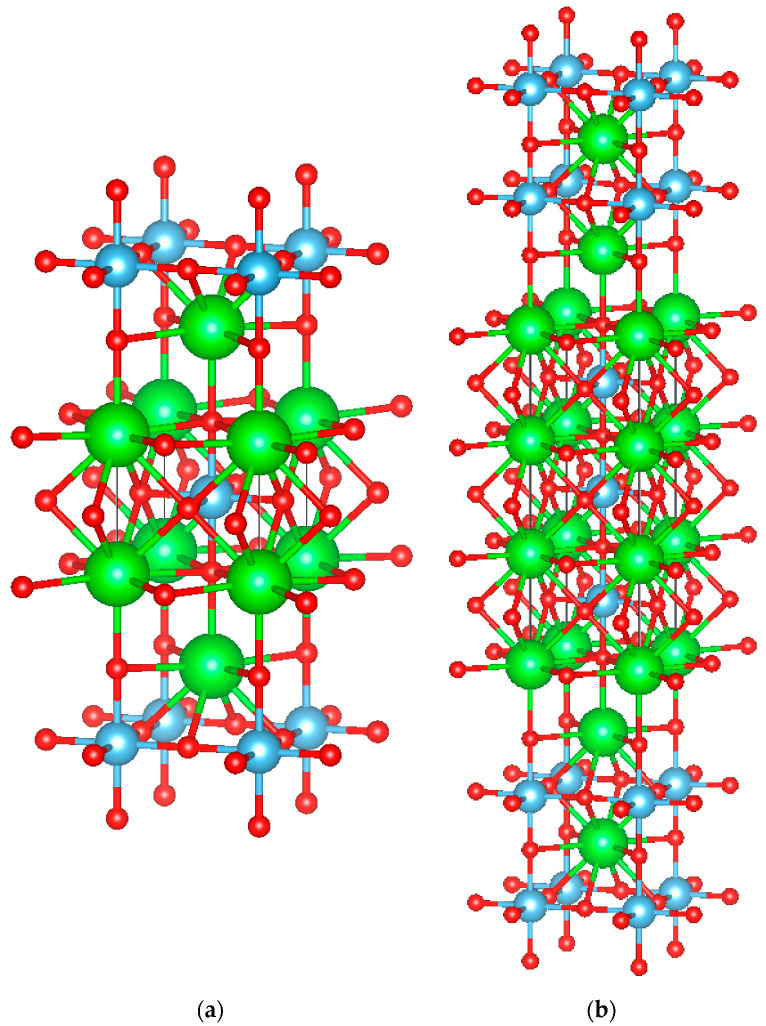
Crystal structure of (**a**) Sr_2_TiO_4_; (**b**) Sr_4_Ti_3_O_10_. Where: large (green) balls—strontium; medium (blue) balls—titanium; small (red) balls—oxygen [31,32].

The R-P phases are the layered perovskite-like oxides with the general formula A_n+1_B_n_O_3n+1_. Their structure consists of nABO_3_ perovskite layers situated between two AO rock-salt layers along the **c** crystallographic direction. The number of perovskite polyhedral units determines the phase peculiarities. The structures of two representatives of this series for our systems, namely Sr_2_TiO_4_ (n = 1) and Sr_4_Ti_3_O_10_ (n = 3), are shown in Figure 5.

Sr-Ti-O phases of this R-P series differ in the length of perovskite-like fragments. The presence of such identical perovskite-like layers suggests that layer alternation defects can quite easily occur in these structures. That is, fragments with one or two (or more) perovskite-like layers joining each other can appear. This may explain the observed violation of periodicity in electron microscopy images.

The formation of periodicity violation defects raises the question of their effect on X-ray powder diffraction patterns and the possibility of calculating their concentrations from diffraction data.

### 3.2. Simulation of XRD Patterns of Sr_2_TiO_4_ Containing Defects of Layer Alternation

Violation of the regular order in the alternation of layers can lead to various diffraction effects that affect the shape of the X-ray diffraction pattern profile. These are, for example, the broadening and/or shift of diffraction peaks with certain Miller indices [33] or the appearance of diffuse scattering maxima [23], among others. A technique that allows us to analyze the type of defects and estimate the concentration of planar defects is based on the simulation of diffraction patterns for one-dimensionally disordered structures [34].

#### 3.2.1. Real Structure Model of Sr_2_TiO_4_

Modeling of the diffraction patterns was carried out using the program [28], which implements the method for constructing a full profile of X-ray diffraction patterns for highly dispersed and partially disordered objects. For the calculations, layers with the SrTiO_3_ perovskite structure (layer A) and with the SrO structure (layer B) were chosen as “building blocks.”

The ideal structure of Sr_2_TiO_4_ was set as a model with strict alternation of layers A and B (the fractions of layers *W_A_ = W_B_* = 0.5, conditional probability of the existence of layer B after layer A *P_AB_* = 1, as well as the probability of the existence of layer A after layer B *P_BA_* = 1, which means that the probabilities *P_AA_* = 0 and *P_BB_* = 0). 

An imperfect structure implies the appearance of layer A after layer A with some probability. Therefore, the fraction of layers A should be more than half. Thus, the structure with planar defects was specified as a particle of the same size but with the following conditions:

- the fraction of layer A *W_A_* = 0.5 + δ, and layer B, in turn—*W_B_* = 0.5 − δ; here δ is varied parameter;
- the probability of occurrence of layer B after layer B *P_BB_* = 0 (but the probability of occurrence of layer A after layer A *P_AA_* ≠ 0 and depends, obviously, on the value of δ).

With the introduction of such conditions, with a certain probability, particles with an increased, in comparison with the ideal structure, length (thickness) of the perovskite-like layer can appear.

Obviously, the δ parameter is proportional to the defect concentration, which in this case can be defined as the fraction of SrO layers absent in the structure Sr_2_TiO_4_ (or the fraction of SrTiO_3_ layers additionally intercalated into the structure).

Specific parameters of the model used for calculations (lattice parameters, layer thicknesses, atomic coordinates, particle sizes) are given in Table S3.

#### 3.2.2. Results of Simulation of Diffraction Patterns

**Figure 6:**
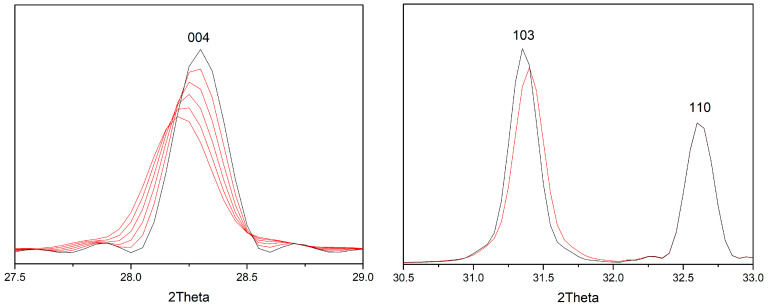
Some X-ray diffraction peaks calculated for Sr_2_TiO_4_ at different values of δ. XRD patterns for perfect (δ = 0) and imperfect structures are shown by black and red colors, respectively (δ value from 0 to 0.01 for 004 peaks, and δ = 0.01 for 103 and 110 peaks).

The calculated diffraction patterns (several characteristic reflections taken as an example) obtained during the simulation are shown in Figure 6.

According to the simulation data (Table 1), it can be seen that in the presence of defects, reflections from the different families of planes behave differently. The positions of some peaks shift while others remain in their place, determined only by the average parameters of the lattice. Peaks with zero indices l, such as 110, 200, and 220, do not change their positions, while reflections with a nonzero index l shift, some of which are very noticeable (for a given value of δ). In particular, the 004 peak shifts to the smaller angles and broadens (Figure 6), as observed in the experimental X-ray diffraction pattern of sample 2 (Figure 2b).

The simulation results show that our test samples can have different concentrations of defects. Accordingly, the diffraction patterns were simulated using the program [33] for samples under study by varying the parameter δ, lattice parameters, and atomic coordinates. The results of the model optimization are shown below.

#### 3.2.3. Compliance of the Calculated Diffraction Pattern with Experimental Data

**Figure 7:**
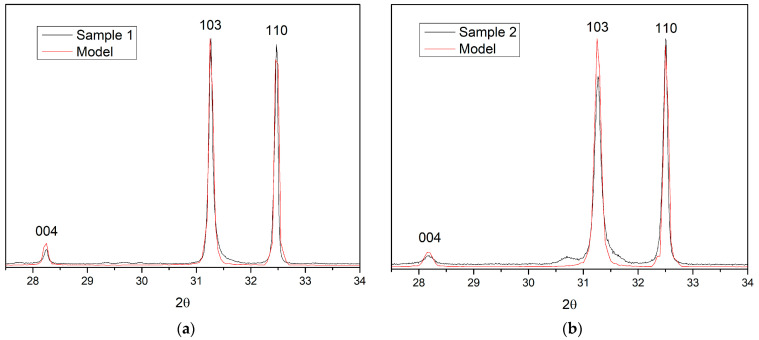
Comparison of experimental and simulated δ = 0.003 x-ray diffraction patterns for samples 1 (**a**) and 2 (**b**) (with δ =0 and δ = 0.003, respectively).

Figure 7 demonstrates characteristic fragments of both samples’ experimental and calculated X-ray diffraction patterns. For sample 1, the XRD pattern calculated based on the model of the perfect crystal structure of Sr_2_TiO_4_ (δ = 0) corresponds well with the experimental data (Figure 7a). For sample 2, the best agreement with the experiment is achieved at δ = 0.003 (Figure 7b). The calculated position of the 004 peaks for the defect structure model coincides with the experimental one and the positions of other peaks. Deviations of the maxima of the theoretical peaks from the experimental ones are within 0.01–0.02° (Table 2), which corresponds to the experimental error in determining the positions of the peaks (see the position of diffraction peaks for sample 1 in Table S2). 

When optimizing the model, we first paid attention to the positions of the diffraction peaks since the preliminary calculations showed that the shifts of some peaks were a characteristic indicator of the presence of defects in the violation of the alternation of layers. Perhaps in the future, it is necessary to make a complete analysis of the influence of these defects on the intensity of maxima. Qualitatively, it is seen that when these defects are introduced, the ratios of intensities of some reflections change, and they become closer to the experimentally observed ones. Still, a detailed analysis of this effect remains to be done. This may be important if we assume that the structure also contains point defects (vacancies). The number of point defects can be judged from the ratio of the intensities of the diffraction peaks. But planar defects also affect intensities. Only taking into account the contribution of planar defects, the number of which can be independently estimated, as our work shows, from the shift of peaks, we should proceed to the analysis of point defects if we are talking about their determination from diffraction data. Let us return once again to the nature of the detected defects. Since we are talking about a decrease in the relative number of layers of the SrO type in the defective crystal structure, this leads to a violation of the stoichiometry of the resulting phase. As for the calculation of the chemical composition of the phase containing defects, it is based on the general formula normalized to the Ti content (=1):

[(0.5 − δ)SrO + (0.5 + δ)SrTiO_3_]/(0.5 + δ) = SrO − [2δ/(0.5 + δ)]SrO + SrTiO_3_.

It is immediately clear here that the difference from the stoichiometry of Sr_2_TiO_4_ is −[2δ/(0.5 + δ)]SrO. For δ = 0.003, the difference from stoichiometry in Sr and O is −0.012. 

Strontium ions that are not included in the crystal structure segregate on the surface, modifying their composition and properties. It is important to note that a number of authors [7,8,9,10] associate the catalytic activity of Sr_2_TiO_4_ oxides in the oxidative coupling of methane precisely with the segregation of Sr on the particle surface.

Since peak positions are of such great importance in terms of determining the concentration of defects, it is obvious that it is desirable to use precision experimental data obtained using synchrotron sources. At a low defect concentration (about 1% as in our case), the deviations of the position of the peak do not exceed 0.1°, which, of course, requires special attention to measurement accuracy.

## 4. Conclusions

In this work, a crystal-chemical analysis of the possible structure of planar defects has been performed, defect structures were simulated, and the effects of such defects on the X-ray diffraction patterns of oxides of the A_2_BO_4_ type were established using Sr_2_TiO_4_ as an example. For the calculations, we used the method of constructing probabilistic models of one-dimensionally disordered structures. The most general qualitative results in this work, related to A_2_BO_4_ (R-P) oxides of any chemical composition, consisted of determining the diffraction signs of the presence of this type of defects, namely, the shift of peaks with specific Miller indices (see Table 1) with respect to their positions determined using the average lattice parameters. Of course, to obtain quantitative estimates, simulations must be carried out for each specific compound, taking into account specific lattice parameters and specific compositions.

A relation has been established between the concentration of planar defects and the real chemical composition (nonstoichiometry) of the Sr_2_TiO_4_ phase. The presence of defects leads to the Ti enrichment of particle volume and, consequently, to the enrichment of the surface with Sr. The latter can explain the catalytic activity of Sr_2_TiO_4_ in the oxidative coupling of methane.

## Figures and Tables

**Table 1:** Position of diffraction maxima.

2ϴ (°) without Defects, δ = 0	2ϴ (°) with Defects, δ = 0.01	h	k	l	Δ(2ϴ) (°)
23.90	23.88	1	0	1	−0.02
28.20	28.11	0	0	4	−0.09
31.25	31.29	1	0	3	+0.04
32.51	32.51	1	1	0	0.00
35.55	35.57	1	1	2	+0.02
42.96	42.87	0	0	6	−0.09
43.5	43.56	1	1	4	+0.06
46.64	46.64	2	0	0	0.00
53.06	53.07	2	1	1	+0.01
54.89	54.81	1	1	6	−0.08
55.35	55.39	2	0	4	+0.04
56.02	56.07	1	0	7	+0.05
57.24	57.21	2	1	3	−0.03
65.26	65.19	2	0	6	−0.07
68.08	68.08	2	2	0	0.00

**Table 2:** The positions of diffraction peaks for sample 2 calculated using the Rietveld method (average lattice parameters) and by modeling diffraction patterns for one-dimensionally disordered structures (at δ = 0.003).

h	k	l	2ϴ (°)			Δ(2ϴ) (°) (Experiment-Rietveld Model)	Δ(2ϴ) (°) (Experiment-Defect Model)
			Experiment	Rietveld Model	Defect Structure Model		
1	0	1	23.87	23.91	23.89	−0.04	−0.02
0	0	4	28.17	28.23	28.17	−0.06	0.00
1	0	3	31.27	31.27	31.27	0.00	0.00
1	1	0	32.49	32.51	32.51	−0.02	−0.02
1	1	2	35.55	35.56	35.55	−0.01	0.00
0	0	6	42.89	42.91	42.90	−0.02	−0.01
1	1	4	43.55	43.58	43.55	−0.03	0.00
2	0	0	46.63	46.64	46.64	−0.01	−0.01
2	1	1	53.06	53.07	53.06	−0.01	0.00
1	1	6	54.85	54.85	54.84	0.00	0.01
2	0	4	55.39	55.41	55.38	−0.02	0.01
1	0	7	56.05	56.12	56.07	−0.07	−0.02
2	1	3	57.24	57.23	57.22	0.01	0.02
2	0	6	65.22	65.22	65.22	0.00	0.00
2	2	0	68.08	68.09	68.08	−0.01	0.00

## Data Availability

Data available upon request.

## Supplementary Materials

The supporting information can be downloaded at: https://www.mdpi.com/article/10.3390/ma15217642/s1.
