# Supplementary material for: Nonstoichiometry Defects in Double Oxides of the A2BO4-Type
Source: Materials (Basel). 2022 Oct 31;15(21):7642. doi: 10.3390/ma15217642 (PMC9654644; doi:10.3390/ma15217642)
Supplement: Supplementary file 1 [file materials-15-07642-s001.zip › materials-1978134-supplementary.pdf]

Supplementary

This file contains:

One figure:

Figure S1: Experimental and model (according to the results of refinement by the Rietveld method) diffraction patterns: (*a*) Sample 1; (*b*) Sample 2.

Three tables:

Table S1: Structural parameters obtained from GSAS-II. Space group of symmetry I4/mmm.

Table S2: Positions of diffraction peaks for sample 1 calculated from the results of modeling by the Rietveld method (average lattice parameters) and by the method of modeling diffraction patterns for one-dimensionally disordered structures (δ = 0)

Table S3: Optimized Model Parameters for Samples 1 and 2

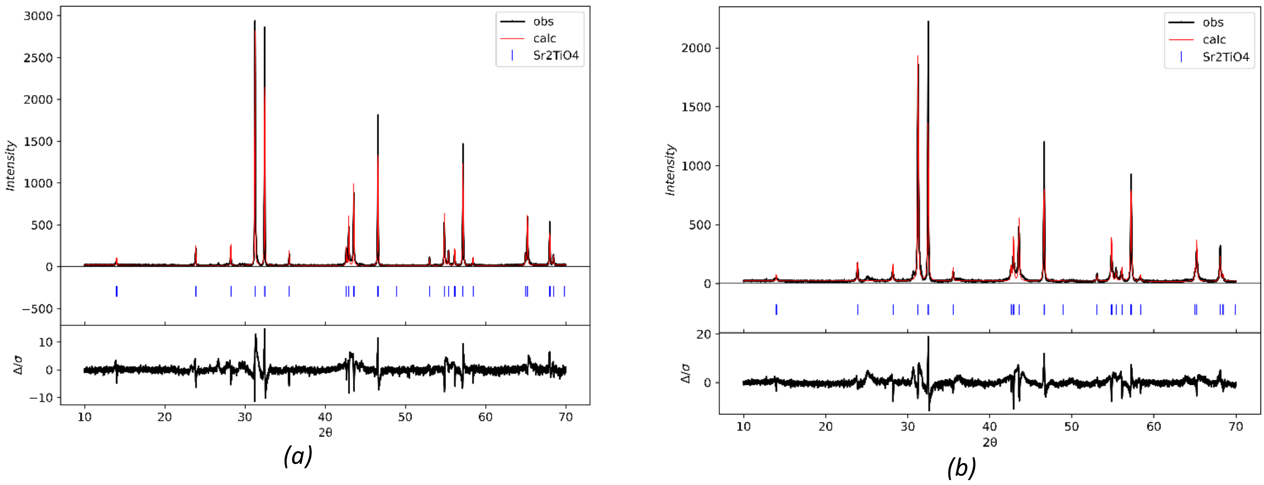

(*a*) (*b*)

Figure S1. Experimental and model (according to the results of refinement by the Rietveld method) diffraction patterns: (*a*) Sample 1; (*b*) Sample 2.

Table S1. Structural parameters obtained from GSAS-II. Space group of symmetry I4/mmm.

Sample 1				Sample 2			
a (Å)	3.8860(0.0003)			3.8823(0.0002)			
c (Å)	12.5910(0.0008)			12.6059(0.002)			
Coordinates							
Atom	x	y	z	Atom	x	y	z
Sr	0	0	0.35320	Sr	0	0	0.35216
Ti	0.5	0.5	0.5	Ti	0.5	0.5	0.5
O1	0.5	0	0.5	O1	0.5	0	0.5
O2	0	0	0.15728	O2	0	0	0.15873
Thermal parameters							
Atom	U			U			
Sr	0.00523			Sr	0.01538		
Ti	0.00005			Ti	0.00005		
O1	0.01222			O1	0.02015		
O2	0.01105			O2	0.05182		
R (%)	20.12			21.02			

Table S2. Positions of diffraction peaks for sample 1 calculated from the results of modeling by the Rietveld method (average lattice parameters) and by the method of modeling diffraction patterns for one-dimensionally disordered structures (δ = 0)

h	k	l	2Θ (°)			Δ(2Θ) (°) (Experiment - Rietveld Model)	Δ(2Θ) (°) (Experiment - Defect Model)
			Experiment	Optimized Model	Rietveld Model		
1	0	1	23.86	23.88	23.89	-0.02	-0.01
0	0	4	28.24	28.23	28.26	-0.02	0.00
1	0	3	31.27	31.27	31.27	0.00	0.00
1	1	0	32.46	32.47	32.48	-0.02	-0.01
1	1	2	35.53	35.53	35.53	-0.01	-0.01
0	0	6	42.95	42.96	42.96	-0.01	-0.01
1	1	4	43.57	43.56	43.58	-0.02	0.01
2	0	0	46.58	46.59	46.59	-0.01	-0.01
2	1	1	53.01	53.01	53.02	-0.01	0.00
1	1	6	54.87	54.87	54.87	0.00	0.00
2	0	4	55.38	55.37	55.39	-0.01	0.01
1	0	7	56.15	56.14	56.17	-0.02	0.00
2	1	3	57.19	57.18	57.19	0.01	0.01
2	0	6	65.23	65.23	65.23	0.00	0.00
2	2	0	68.01	68.01	68.02	-0.01	0.00

Table S3. Optimized Model Parameters for Samples 1 and 2

	Sample 1	Sample 2	
Lattice parameters (Å)			
a =	3.8866	3.8826	
c =	12.5961	12.6176	
Layer A			
Layer thickness (Å)	3.6755	3.7633	
Coordinates			
Atom	x	y	z
Sr	0	0	0
Ti	0.5	0.5	0.5
O1	0	0.5	0.5
O2	0.5	0	0.5
O3	0.5	0.5	0
Layer B			
Layer thickness (Å)	2.6225	2.5455	
Coordinates			
Atom	x	y	z
Sr	0	0	0
O	0.5	0.5	0
Particle size (Å), cylinder shape			
Height	865	490	
Diameter	1100	865	
